# Neural correlates of verbal recognition memory in obese adults with and without major depressive disorder

**DOI:** 10.1002/brb3.1848

**Published:** 2020-09-20

**Authors:** Maria R. Restivo, Geoffrey B. Hall, Benicio N. Frey, Margaret C. McKinnon, Valerie H. Taylor

**Affiliations:** ^1^ Women’s College Research Institute Women’s College Hospital Toronto ON Canada; ^2^ Department of Psychology, Neuroscience & Behaviour McMaster University Hamilton ON Canada; ^3^ Department of Psychiatry and Behavioural Neurosciences McMaster University Hamilton ON Canada; ^4^ Department of Psychiatry University of Calgary Calgary AB Canada

**Keywords:** cognition, depression, fMRI, memory, neural activation, obesity

## Abstract

**Background:**

Obesity and major depressive disorder (MDD) independently contribute to memory impairment. Little is known about shared neural mechanisms that may result in the cognitive impairment experienced by these populations. This study's aim was to determine how obesity impacts neural activity during a verbal recognition memory task in individuals both with and without MDD.

**Methods:**

Functional magnetic resonance imaging was employed to examine whether differences in neural activation patterns would be seen across three groups during the Warrington's Recognition Memory Test. Three study groups are reported: 20 subjects with obesity but without MDD (bariatric controls), 23 subjects with past or current MDD and obesity, and 20 normal BMI controls (healthy controls).

**Results:**

Three‐group conjunction analyses indicated that overlapping neural regions were activated during both encoding and retrieval processes across all groups. However, second‐level 2‐group t‐contrasts indicated that neural activation patterns differed when comparing healthy and bariatric controls, and when comparing bariatric controls and bariatric MDD participants.

**Discussion:**

Results indicate that obesity in conjunction with MDD confers a subtle impact on neural functioning. Given high rates of obesity and MDD comorbidity, and the role of cognition on ability to return to premorbid level of functioning, this association should inform treatment decisions.

## Graphical abstract

Obesity and major depressive disorder (MDD) independently contribute to memory impairment. This study's aim was to determine how obesity impacts neural activity during a verbal recognition memory task in individuals both with and without MDD. Results indicate that obesity in conjunction with MDD confers a subtle impact on neural functioning.

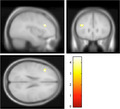

## SIGNIFICANT OUTCOMES

1. Both obesity and depression are associated with neural activation changes. When the two illnesses are combined, there is risk of additional cognitive issues occurring.
2. It is important to recognize risk factors for weight gain in individuals with depression and to ensure treatments are provided to minimize these risks.

## LIMITATIONS

1. A limitation of the study is the lack of an additional fourth group of individuals with MDD who do not have obesity, as it would allow us to further elucidate the potential additive and independent effects of MDD and obesity on cognition.
2. As our study is cross‐sectional, it cannot speak to the causality of the associations seen between obesity, depression, and neural activation patterns.

## 1 INTRODUCTION

Major depressive disorder (MDD) and obesity are among the leading causes of disability worldwide (Ferrari et al., 2013; Ng et al., 2013). Although it is well established that obesity is associated with cardiovascular disease (CVD) risk factors such as type II diabetes (T2D) and hypertension, obesity's detrimental impact on brain health has become increasingly recognized in recent years (Boots et al., 2019; Martins, Monteze, Calarge, Ferreira, & Teixeira, 2019; Milaneschi, Simmons, van Rossum, & Penninx, 2019; van den Berg, Kloppenborg, Kessels, Kappelle, & Biessels, 2009). The deleterious effects of obesity on cognitive functioning have been demonstrated across a variety of cognitive domains, most reliably in the areas of memory, attention, and executive function (Alosco et al., 2013; Boeka & Lokken, 2008; McIntyre et al., 2018; Prickett, Brennan, & Stolwyk, 2015). Notably, changes in memory recollection and executive function are also among the most consistently reported deficits in patients with MDD in both depressive and euthymic states (Alves et al., 2014; Degl'Innocenti & Backman, 1999; Drakeford et al., 2010; Gorwood, Corruble, Falissard, & Goodwin, 2008; MacQueen, Galway, Hay, Young, & Joffe, 2002). Rarely, however, is weight or body mass index (BMI) controlled for in studies examining cognition in MDD (Amiri, Behnezhad, and Nadinlui 2018; Mansur, Brietzke, & McIntyre, 2015). More recently, studies have begun to explore the shared neural mechanisms that may contribute to cognitive impairment seen in both obese and depressed populations (Milaneschi et al., 2019).

Functional magnetic resonance imaging (fMRI) has been used extensively in identification of abnormal neural circuitry underlying cognitive deficits observed in patients with MDD, but very few studies have used fMRI to investigate these deficits in a population with obesity (Carnell, Gibson, Benson, Ochner, & Geliebter^2012^; Michaelides, Thanos, Volkow, & Wang,^2011^). Instead, the majority of fMRI work done with respect to obesity has been focused on appetite regulation, eating behavior, and reward circuitry (Stanek, Smith, J., & Gunstad, 2011). More robustly, increased BMI has been associated with unique structural brain changes in patients with both MDD and bipolar disorder (BD) (Bond, Gigante, Torres, Lam, & Yatham, 2011; Bond et al., 2014; Shinsuke et al., 2018), including gray matter and white matter volume reductions in frontal, temporal, and subcortical limbic regions. These areas are thought to be important in memory processing, as well as being implicated in both BD and MDD pathophysiology (Diener et al., 2012; Konarski et al., 2008). However, no study to date has investigated the impact of obesity on memory using an fMRI task activation paradigm.

The primary aim of the current study, therefore, was to determine how obesity impacts neural activation patterns during a verbal recognition memory task in a sample of individuals with obesity, with and without comorbid MDD. We sought to examine whether the differences in neural activation during memory processes in MDD patients could in part be etiologically linked to obesity using a recognition memory paradigm under fMRI. We hypothesized that obesity would alter neural activation during performance in regions involving executive function (such as the prefrontal cortices) and that the additional presence of MDD would further alter this neural activation pattern.

## 2 METHODS

### 2.1 Subjects

This study was conducted at St. Joseph's Healthcare, Hamilton, Canada, and received full ethics approval (HIREB 09‐3254) (Restivo, McKinnon, Frey, Hall, & Taylor, 2016). Subjects were placed into three groups: healthy controls (normal BMI, no psychiatric diagnosis) [HC], bariatric controls (obese, no psychiatric diagnosis) [BC], and bariatric MDD subjects (obese, current, or past diagnosis of MDD) [B‐MDD]. All bariatric subjects were enrolled in the bariatric surgery program at St. Joseph's Healthcare Hamilton at the time of recruitment. *Inclusion criteria* for all groups were as follows: age 18–60 years, ability to provide informed consent, and native English speaker (or having learned English by age 6). Additionally, HCs were required to have a BMI between 18.5 and 24.9 (normal range). *Exclusion Criteria* included the presence of a current or pre‐existing neurological condition (e.g., epilepsy and severe head trauma) or unstable and/or severe medical condition (e.g., cancer and severe heart attacks), contraindications to MRI, left‐handedness (confirmed via Edinburgh Handedness Inventory), having been administered any of the cognitive study measures within the past 12 months, a history of a confirmed learning disorder or developmental disability diagnosis (e.g., attention‐deficit/hyperactivity disorder) or a Full Scale Intelligence Quotient (FSIQ) < 70, an inability to complete the testing (e.g., due to a hearing or vision impediment), the presence of alcohol or substance abuse within the last 6 months or lifetime dependency, and having been administered electroconvulsive therapy (ECT) within the last 24 months. In addition, the presence of a past or current psychiatric condition was an exclusion criterion for both the HC and BC groups. Full recruitment procedures and detailed study protocol are outlined in Restivo et al. (2016).

Twenty‐one HCs, 25 BCs, and 23 B‐MDD participants consented and enrolled in the study. Six subjects (one HC and five BCs) were unable to complete the neuroimaging testing due to feelings of claustrophobia and anxiety. Subjects were age‐matched across all three groups, resulting in 20 HCs, 20 BCs, and 23 B‐MDDs (20 age‐matched B‐MDD subjects as well as an additional three B‐MDD subjects who also completed the study at the time of publication for a total of *n* = 23 in the B‐MDD group). Psychiatric diagnoses were evaluated via the Structured Clinician Interview for DSM‐IV (SCID‐I) (First, Williams, Spitzer, & Gibbon, 2007). Both depressed and euthymic patients with a past diagnosis of depression were recruited into the B‐MDD group.

### 2.2 Demographical, medical, and psychiatric characteristics

An extensive list of corollary information was also obtained. Data from administered standardized questionnaires, clinical interviews, and subject charts and medication profiles were collected in order to identify and control for potential confounders. For full details of the various metrics included, please see the protocol published by Restivo et al. (2016). Exploratory descriptive group analyses were performed to investigate and characterize group means, ranges, and standard deviations. One‐way between‐group analysis of variance (ANOVA) tests were performed on all continuous covariates of interest. Chi‐square analyses were run to compare group differences in categorical variables. Significant ANOVA test results were then further investigated by means of pairwise comparisons (Tukey's HSD). Proportion of comorbidities (e.g., hypertension) was compared between bariatric patient groups to ensure that one group was not heavily loaded with potential confounders in order to control for potential group differences. Additionally, comorbidity variables considered potential confounders were explored further in secondary analyses of ANOVA models found to be significant. Pearson's product–moment correlation coefficients between cognitive outcomes and medication load composite scores were also calculated.

#### 2.2.1 Medical

Extensive demographical, medical health (e.g., cardiovascular comorbidities), and psychiatric information was collected for all subjects. Anthropomorphic data collected included weight, height, BMI, waist and hip circumferences, average systolic and diastolic blood pressures, a random glucose “finger prick test” value, lipid profile values, and hemoglobin HbA1c values. The presence of type II diabetes (T2D), hypertension, and dyslipidemia was determined by data extraction of patients’ medical charts. Obstructive sleep apnea (OSA) was determined via the Berlin Sleep Questionnaire (Chung et al., 2008). Subjects were coded as high risk or low risk for OSA (subjects whose OSA was being currently treated and controlled by a continuous positive airway pressure ventilator were coded as low risk). Nutritional intake was assessed via a 3‐day dietary record (Food Frequency Questionnaire [FFQ]), with one day being from the weekend (Willett & Leibel, 2002); both total daily caloric intake and diet component analyses were also completed. Subjects were asked to provide a complete listing of current medications, vitamins, and herbal supplements (including dosage and indication) during their first study visit; medication history was also confirmed via data extraction of bariatric patients’ medical charts and recorded clinic staff encounters. Following previously employed methodology by Sackeim (2001) and Hassel et al. (2008), we generated a composite measure of total (psychotropic) medication load based on dosage and medication class for each B‐MDD subject.

#### 2.2.2 Demographic

Age at time of neuropsychological testing, years of education, sex, and ethnicity were collected for each subject. The Cognitive Failure Questionnaire (CFQ) (Broadbent, Cooper, FitzGerald, & Parkes, 1982) was used to assess subjective feelings of cognitive dysfunction, and the Sheehan Disability Scale (SDS) was used to assess functioning across three life domains (work/school, social life, and family life/home responsibilities) (Sheehan, Harnett‐Sheehan, & Raj, 1996).

#### 2.2.3 Psychiatric

Mood‐rating questionnaires on or near the day of neuroimaging were administered. The Beck Depression Inventory (BDI) (Beck, Steer, & Brown, 1996) and Hamilton Rating Scale for Depression‐17 (HAM‐D‐17) (Hedlung & Vieweg, 1979) were used to measure the presence of depressive symptoms, while the Altman Rating Scale for Mania (ARSM) (Altman, Hedeker, Peterson, & Davis, 1997) and Young Mania Rating Scale (YMRS) (Young, Biggs, Ziegler, & Meyer, 1978) were used to measure for the presence of mania symptoms.

Analyses for potential group differences in nutritional intake, medication load, and comorbid illnesses (such as hypertension, type II diabetes, and obstructive sleep apnea risk) were completed.

### 2.3 fMRI task paradigm and procedures

We designed a recognition memory paradigm based on a standardized neuropsychological measure, adapted for use in an MRI. We used a fixed block design, word encoding, and recognition paradigm. Subjects performed an MRI version of Warrington's Recognition Memory Test (Warrington, 1984) (word subtest only) to assess material‐specific memory deficits in adults. Subjects initially underwent a practice session outside the scanner on the day of MRI testing. During the encoding task, subjects were presented with a 50‐item target word list and asked to indicate whether their association with the word was “pleasant” or “unpleasant” by pushing a response pad with their left or right index finger, respectively. Target words were considered emotionally neutral (subject choice is arbitrary). Following completion of the encoding task, subject recognition memory for target words was assessed immediately. During the recognition subtask, subjects were presented with a pair of word stimuli; the previously seen encoding target word was paired with a similar distracter word (also taken from the original task). Subjects were asked (in a forced‐choice paradigm) to indicate whether the encoding target word appeared on the left or right side of the screen by pressing buttons on a response pad with their left or right index finger. Words were randomized to appear on each side 50% of the time. For both subtasks, stimuli were presented at a rate of 3 s each in 10‐item blocks (activation condition) and alternated with 21 s of a rest condition. In order to meet the requirements of working with a bariatric population, a rear‐projection display system was engineered at the Imaging Research Centre at St. Joseph's Healthcare Hamilton. Responses were recorded by E‐Prime and exported to Statistical Package for the Social Sciences (SPSS) for statistical analysis (IBM Corp, 2013).

### 2.4 Image acquisition

Scanning was performed on a General Electric 3 Tesla whole‐body short‐bore scanner with eight parallel receiver channels (Milwaukee, WI). For each subject, functional images were collected using a T2* interleaved echo‐planar imaging sequence: 41 axial slices, flip angle = 60°, TE = 35 ms, TR = 3,000 ms, FOV = 24 cm, matrix frequency = 128, phase = 64; slice thickness = 3 mm, and no skip. T2 images were coregistered to images acquired from a T1‐weighted anatomical scan (3D‐SPGR pulse; IRP sequence; matrix frequency = 512 x 512, flip angle 90^0^; TE = 1.32 ms; TR = 6.228 ms; TI = 900ms; FOV = 24 cm; 1‐mm axial slices; and no skip).

### 2.5 Image preprocessing

Preprocessing (slice‐time correction, motion correction, spatial normalization, and smoothing) was performed using SPM12 (http://www.fil.ion.ucl.ac.uk/spm/) in MATLAB 8.3.0 (The MathWorks, Inc., 2012). The first three volumes of each subtask were removed as dummy volumes. The 81 volume images of each task were realigned to the first image of the time series and transformed to Montreal Neurological Institute (MNI) space as defined by the SPM12 T1 template. Following motion correction in SPM, subject head motion was examined manually for movement greater than 3 mm in any axis direction. All subjects were within the 3‐mm threshold. Following this, ArtRepair (http://cibsr.stanford.edu/tools/human‐brain‐project/artrepair‐software) was run on individual subjects to further correct head movement differences across consecutive volumes. Volumes following movement greater than 0.5 from the previous movement were considered artifacts and removed (deweighted) from the dataset. Functional datasets were smoothed using a full‐width half‐maximum Gaussian filter of 5 mm.

A time‐series model was constructed based on alternating periods of activation and rest using and modeling the hemodynamic response. A general linear model approach for time‐series data was used to identify significantly activated voxels. A contrast matrix testing for signification activation was defined as the encoding condition versus the rest condition and as the retrieval condition versus the rest condition. Within‐group t‐statistics were calculated as standardized z‐scores in projection maps. Threshold for significant activation (uncorrected) was *p* < .001. In order to identify regions common to all three groups, we conducted a conjunction (conjunction null hypothesis) of the three subject groups across activation > rest task conditions. Statistical maps for conjunction activation were FWE‐corrected at *p* = .05.

### 2.6 Behavioral task—statistical analysis

Functional imaging data were analyzed using SPM12. Percentage of words coded as pleasant or unpleasant during the encoding subtask was compared across groups using one‐way between‐group ANOVA comparisons. Raw correct number of words remembered in the recognition task was calculated per subject, along with correct percentage of responses (adjusted to represent percentage of responses where subject responded). Response time (corrected to exclude null responses) was calculated for each subject and subtask; response time differences were contrasted across groups using one‐way between‐group ANOVAs. Behavioral data were verified to be normally distributed prior to running ANOVAs.

## 3 RESULTS

### 3.1 Subject demographics

Our final sample consisted of 63 subjects, 60 of whom were age‐matched in three groups of 20 subjects (an additional three subjects who completed all study testing are included in the final B‐MDD group as well). Groups did not vary in sex, ethnicity, or Full Scale IQ (*p* < .05) (see Table 1) (though HC subjects did complete more years of education versus BC subjects and B‐MDD subjects [*p* < .05]). Medical characteristics (such as BMI, comorbidity diagnosis, and medication load) and nutritional intake did not differ significantly between the BC and B‐MDD groups. As expected, group differences were seen in subjective ratings of overall disability/impairment (as rated by the SDS measure) and cognitive impairment (as rated by the CFQ), with the B‐MDD group reporting the highest level of impairment on both measures (*p* < .05). Scores on the self‐reported BDI measure indicated an average level of mild depression in the B‐MDD group (*M* = 17.2 [10.1]); BDI scores differed significantly across groups (*F*[2,59] = 18.09, *p* < .001). In addition, a Tukey–Kramer post hoc analysis indicated that the BC group had a significantly higher (*p* = .01) BDI score (*M* = 9.3[9.2]) than the HCs (*M* = 1.7 [4.4]); however, this score still fell below the scale threshold for clinical depression. BDI and HAM‐D scores at the time of testing were entered in an exploratory ANOVA as independent variables, with behavioral measure outcomes as dependent variables (as detailed below).

**Table 1:** Study sample characteristics

	Healthy Controls (HC) (*n* = 20)	Bariatric Controls (BC) (*n* = 20)	Bariatric MDD (MDD) (*n* = 23)
Age (Mean, *SD*)	43.8 (11.0)	43.9 (10.7)	42.6 (11.0)
Sex (Male:Female)	2:18	1:19	2:21
Years of Education^a^	16.1 (2.3)	14.5 (2.2)	14.1 (2.3)
Ethnicity (Caucasian %)	85.0	93.8	78.9
BMI	22.4 (2.0)	44.7 (3.2)	43.8 (4.7)
Weight (kg)	60.3 (7.1)	121.5 (11.5)	116.6 (15.1)
Height (cm)	164.0 (8.3)	165.2 (4.2)	163.0 (6.5)
Waist Circumference (cm)	74.6 (5.2)	121.4 (10.1)	124.9 (11.8)
Hip Circumference (cm)	97.7 (5.8)	140.3 (8.4)	134.4 (11.6)
Hypertension^b^ (%)	0.0	40.0	39.1
Average Systolic BP^c^ (mmHg)	119.8 (9.3)	135.6 (16.1)	133.6 (12.3)
Average Diastolic BP^c^ (mmHg)	74.6 (16.2)	78.4 (6.7)	76.2 (11.7)
Average Heart rate^c^	73.4 (12.8)	83.4 (12.3)	80.3 (10.2)
TDII (%)^d^	0.0	25.0	22.7
Ha1bc	n/a	0.060	0.059
Random Glucose Test	5.8 (1.6)	5.9 (2.6)	5.2 (0.8)
Hyperlipidemia^e^	0.0	25.0	36.4
Total Cholesterol	n/a	4.55 (0.95)	4.81 (0.95)
HDL	n/a	1.32 (0.3)	1.18 (0.30)
LDL	n/a	2.61 (0.69)	2.96 (0.87)
Triglycerides	n/a	1.39 (0.80)	1.60 (0.60)
OSA Risk (%High Risk)	0.0	45.0	69.6
FSIQ (WASI)	112.2 (13.4)	107.9 (11.7)	104.8 (12.1)
HAM‐D^a^, ^b^	1.6 (2.9)	1.6 (1.9)	6.7 (4.2)
BDI^a^, ^b^	1.9 (5.6)	9.3 (9.2)	17.2 (10.1)
YMRS^a^, ^b^	0.6 (0.9)	0.5 (0.7)	2.5 (2.5)
ASRM	1.6 (2.6)	4.2 (3.5)	2.4 (2.5)
SDS (averaged across domains)^a^, ^b^	0.0 (0.1)	3.0 (2.8)	4.8 (2.7)
CFQ Total^a^, ^b^	22.9 (10.6)	26.1 (6.5)	39.1 (19.8)

^a^ 3‐group one‐way ANOVA (analysis of variance) *p* < .05.

^b^ Borderline hypertension was collapsed into the hypertension group.

^c^ Two independent measures, 1 min apart, were obtained.

^d^ Borderline, well‐controlled, and suboptimally controlled TDII status were collapsed.

^e^ Elevated lipid value status was also included as hyperlipidemia.

### 3.2 Behavioral data analysis

#### 3.2.1 Encoding

Differences across groups in reaction time (correcting for null trials) were examined using between‐group ANOVAs; no significant differences were found (*F*[2,61] = 0.29, *p* = .74). Exploratory analyses were performed in order to evaluate whether group membership was associated with differences in the amount of words rated pleasant versus unpleasant; a significant group effect was found (F[2,61]=3.45, *p* = .04). Percentage of words coded as unpleasant did not correlate with HAM‐D or BDI scores, however.

#### 3.2.2 Recognition

After correcting for null trials, no significant differences between groups were seen in reaction time [*F*(2,61) = 0.88, *p* = .41)]. Analyses were performed for accuracy on words previously encoded as pleasant, accuracy on words previously encoded as unpleasant, and overall word recognition accuracy. Raw scores were converted to percentage correct and adjusted to exclude null trials. When including lack of response as incorrect, a significant group difference was seen on memory retrieval performance [*F*(2,61) = 3.32, *p* = .04]. However, when adjusted to exclude null trials, group differences in total word recognition were no longer significant [*F*(2,61) = 1.41, *p* = .25]. No group differences emerged when correct responses coded previously as pleasant versus unpleasant were examined. BDI and HAM‐D scores were not associated with total correct word recognition trials (regardless of whether null trials were included). Behavioral data are summarized in Table 2.

**Table 2:** Behavioral data for the RMT fMRI task

	HC (*n* = 20)	BC (*n* = 20)	MDD (*n* = 23)
*Reaction time*			
Encoding (ms)	1,316.4 (255.6)	1,275.0 (226.0)	1,264.2 (207.5)
Retrieval (ms)	1,398.5 (196.5)	1,506.4 (208.8)	1,415.2 (272.9)
*Encoding task*			
% Encoded as pleasant	67.9 (11.4)	58.1 (13.1)	62.5 (10.9)
% Encoded as unpleasant	31.5 (11.1)	40.8 (13.1)	37.2 (10.9)
Retrieval task			
Total correct (%)*	83.5 (5.8)	76.1 (10.4)	78.7 (10.6)
Total correct adjusted (%)	92.3 (4.5)	87.8 (11.9)	88.9 (8.7)
Pleasant words correct (%)	82.5 (8.2)	75.5 (10.5)	78.0 (11.1)
Unpleasant words correct (%)	84.4 (12.4)	78.7 (17.7)	80.4 (13.8)

* Significant group effect (*p* = .043).

### 3.3 fMRI analysis: encoding

**Figure 1:**
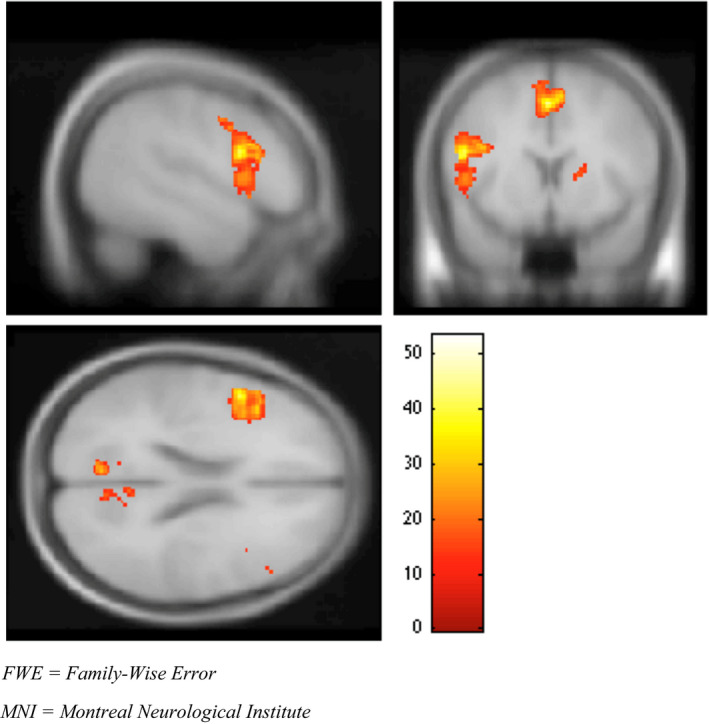
Group effect of diagnosis on encoding > rest contrast at the inferior frontal gyrus (MNI coordinates = −50, 10, 22), *F* = 39, *p*_(FWE corr.)_ = .005. FWE, family‐wise error; MNI, Montreal Neurological Institute

**Figure 2:**
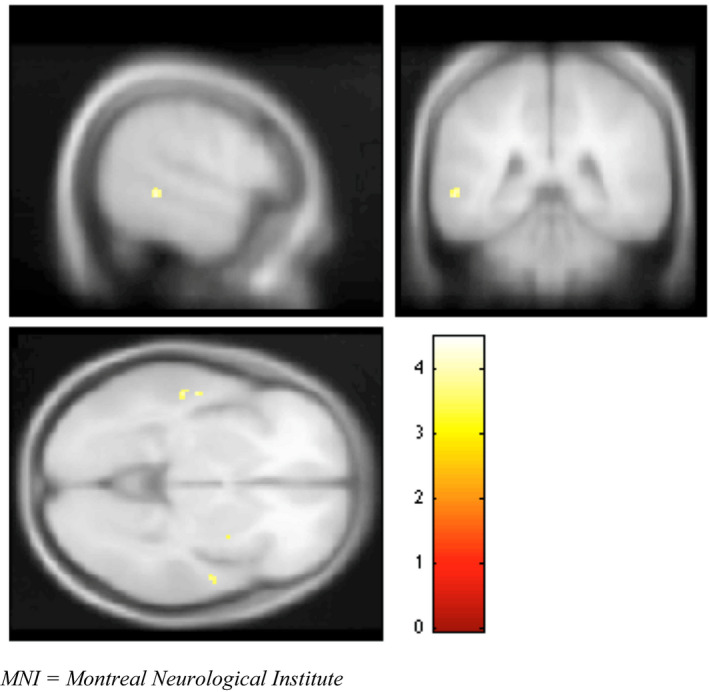
Paired group comparison of encoding > rest activation. BC > HC: (MNI coordinates = −56, −40, −2), *T* = 4.17, *p* < .001 uncorrected. MNI, Montreal Neurological Institute

Whole‐brain conjunction analysis was performed using all three subject groups and creating z‐score spatial maps, in order to investigate regions commonly activated across groups. Using FWE correction, the greatest conjunction analysis activation (group and encoding > rest contrasts) occurred in the inferior frontal (IFG) and medial frontal gyri (MFG) (see Figure 1 and Table 3). Activation was also seen in the posterior cingulate, cuneus, thalamus, and lingual gyrus. Differences in regional activations were further explored via 2‐group t‐contrasts (BC > HC and B‐MDD > BC) (see Tables 4 and 5, and Figure 2). Although overlapping several regions were activated in both t‐contrasts, the BC > HC contrast yielded activation largely centered in the middle and superior temporal gyrus (STG). This pattern differed from that seen in the B‐MDD > BC contrast, which indicated activation in areas of the precuneus (not seen in the BC > HC contrast) and anterior cingulate gyrus. Activations in the middle temporal gyrus (MTG) and insula were also seen in both contrasts.

**Table 3:** Main effect of encoding versus rest condition across groups (conjunction null hypothesis, *p* < .05, FWE whole brain corrected)

Region	Side	BA	MNI Coordinates			*Z* value
			*X*	*Y*	*Z*	
Claustrum	L		−29	21	6	6.16
Inferior Frontal Gyrus	L	47	−40	16	−4	5.59
Medial Frontal Gyrus	L	6	−3	1	49	5.97
Medial Frontal Gyrus	L	6	−4	−5	58	5.92
Superior Frontal Gyrus	L	6	−4	6	59	5.29
Lingual Gyrus	R	18	16	−85	−14	5.75
Inferior Frontal Gyrus	L	9	−48	6	24	5.62
Posterior Cingulate	R	31	8	−55	26	5.47
Posterior Cingulate	R	23	10	−47	25	5.27
Cuneus	L	30	−13	−68	14	5.44
Declive	R		30	−75	−17	5.41
Declive	L		−36	−76	−20	5.31
Claustrum	R		29	23	2	4.94
Precuneus	L	31	−2	−73	24	4.91
Cingulate Gyrus	L	31	−5	−55	31	4.91
Declive	L		−34	−65	−20	4.89
Precentral Gyrus	L	6	−38	2	27	4.89
Cingulate Gyrus	L	31	−11	−40	33	4.89
Inferior Frontal Gyrus	L	9	−49	15	23	4.87

Abbreviations: FWE = family‐wise error; MNI = Montreal Neurological Institute.

**Table 4:** Between‐group (BC > HC) comparisons of the encoding > rest condition (*p* < .001 uncorrected)

Region	Side	BA	MNI coordinates			*Z* value
			*X*	*Y*	*Z*	
Caudate	L		−14	−32	24	4.33
Precentral Gyrus	L	6	−42	−2	45	3.93
Parahippocampal Gyrus	R	19	25	−54	0	3.81
Cuneus	R	23	8	−72	12	3.78
Lingual Gyrus	R	19	15	−66	6	3.49
Superior Temporal Gyrus	R	22	49	−9	−1	3.76
Middle Temporal Gyrus	L	22	−53	−39	−2	3.67
Cingulate Gyrus	L	24	1	−4	29	3.62
Cingulate Gyrus	L	24	1	3	28	3.29
Lingual Gyrus	L	19	−18	−62	2	3.61
Superior Frontal Gyrus	L	6	−3	5	50	3.58
Superior Temporal Gyrus	L	21	−49	−26	−1	3.54
Caudate	R		10	−1	17	3.53
Caudate	R		4	16	17	3.52
Thalamus	L		−18	−9	18	3.52
Thalamus	L		−9	−7	16	3.22
Anterior Cingulate	L	24	−3	21	24	3.51
Medial Frontal Gyrus	R	6	4	0	58	3.48
Declive	R		38	−56	−17	3.47
Insula	R	13	43	−20	1	3.44
Claustrum	R		27	24	9	3.44
Precentral Gyrus	R	9	39	12	35	3.41
Caudate	L		−22	10	17	3.4
Lentiform Nucleus—Putamen	L		−27	−7	−2	3.35
Insula	L	13	−40	−12	17	3.26
Lentiform Nucleus—Putamen	R		29	−2	3	3.23
Lingual Gyrus	R	19	19	−67	0	3.23
Caudate	R		−14	5	17	3.21
Superior Temporal Gyrus	L	22	−51	−5	−2	3.2
Claustrum	R		36	−19	10	3.19
Lingual Gyrus	L	18	−11	−72	6	3.15
Declive	L		−33	−66	−12	3.14
Claustrum	L		−34	−5	3	3.11

Abbreviation: MNI = Montreal Neurological Institute.

**Table 5:** Between‐group (MDD > BC) comparisons of the encoding > rest condition (*p* < .001 uncorrected)

Region	Side	BA	MNI coordinates			*Z* value
			*X*	*Y*	*Z*	
Culmen	L		−25	−52	−21	4.41
Insula	L	13	−40	−13	26	3.57
Middle Frontal Gyrus	L	9	−35	22	27	3.45
Cingulate Gyrus	L	24	−16	2	44	3.4
Inferior Frontal Gyrus	L	45	−45	23	6	3.31
Superior Frontal Gyrus	L	6	−9	11	52	3.29
Middle Temporal Gyrus	L	22	−57	−35	4	3.27
Declive	L		−31	−60	−11	3.25
Parahippocampal Gyrus	L	36	−36	−24	−13	3.23
Caudate	L		−14	−25	30	3.18
Precuneus	L	31	−11	−52	33	3.16
Culmen	R		27	−65	−27	3.13
Anterior Cingulate	L	32	−19	45	12	3.11

Abbreviation: MNI = Montreal Neurological Institute.

### 3.4 fMRI analysis: retrieval

**Figure 3:**
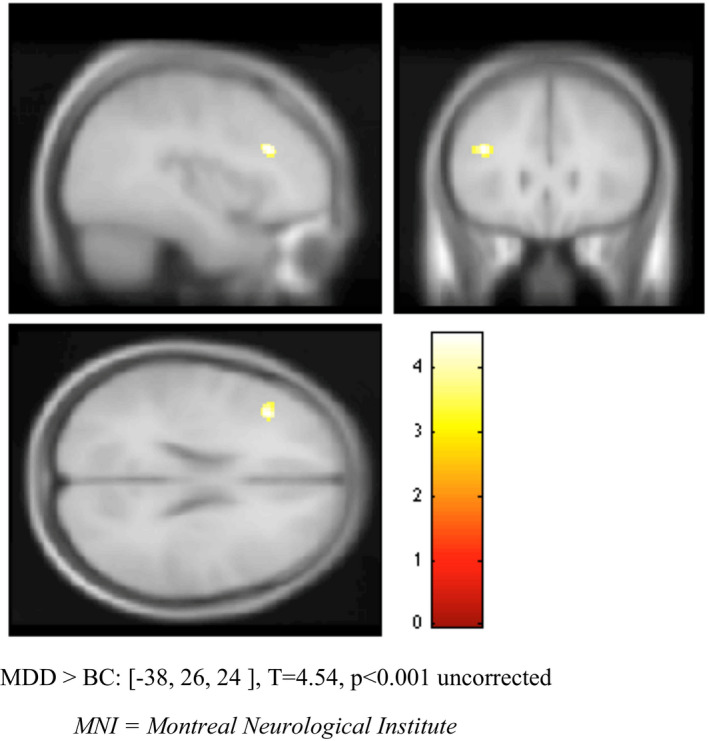
Comparison of dorsolateral prefrontal cortex (BA9) activation between MDD and BC groups. Retrieval > rest activation, *p* < .001 uncorrected. Increased activation in MDD versus BC group shown. MDD > BC: [−38, 26, 24], *T* = 4.54, *p* < .001 uncorrected. MNI, Montreal Neurological Institute

Whole‐brain conjunction analysis was performed using all three study groups in order to investigate regions commonly activated across groups during the retrieval > rest contrast. After applying FWE correction, the greatest number of regional activations was once again seen in the frontal gyrus. Additional activations were seen in the IFG, thalamus, precuneus, posterior cingulate, middle temporal gyrus, cuneus, and cingulate gyrus (see Table 6). BC > HC and B‐MDD > BC contrasts comparing retrieval > rest conditions between groups were also conducted. Although numerous regions of activation were yielded by the BC > HC contrast (listed in Table 7), only two regions of activation were identified in the B‐MDD > BC contrast (the MFG [MNI coordinates: −38, 26, 24, *T* = 4.54, *p*(unc) < .001] and SFG [MNI coordinates: −6, 18, 52, *T* = 3.64, *p*(unc) < .001] (see Table 8 and Figure 3).

**Table 6:** Main effect of retrieval versus rest condition across groups (conjunction null hypothesis, *p* < .05, FWE whole brain corrected)

Region	Side	BA	MNI coordinates			*Z* value
			*X*	*Y*	*Z*	
Medial Frontal Gyrus	L	32	−3	5	48	5.46
Medial Frontal Gyrus	R	6	4	13	43	6.02
Claustrum	L		−31	21	4	5.17
Claustrum	L		−34	13	5	5.71
Declive	R		6	−75	−21	5.46
Thalamus	L	Ventral Anterior Nucleus	−16	−8	14	5.44
Declive	L		−38	−59	−22	5.38
Inferior Frontal Gyrus	L	9	−42	9	28	4.98
Precentral Gyrus	L	6	−42	2	29	5.38
Declive	R		36	−65	−21	5.29
Cingulate Gyrus	L	31	−5	−41	38	5.26
Claustrum	R		31	17	5	5.2
Precentral Gyrus	L	6	−44	−2	32	5.19
Declive	R		30	−77	−19	5.16
Declive	L		−5	−74	−23	5.04
Declive	L		−36	−76	−22	5.02
Cingulate Gyrus	L	31	−5	−37	35	4.89
Cuneus	R	18	26	−69	16	4.88
Precuneus	L	7	−28	−64	30	4.87
Uvula	R		34	−65	−27	6.64

Abbreviations: FWE, family‐wise error; MNI, Montreal Neurological Institute.

**Table 7:** Between‐group (BC > HC) comparisons of the retrieval > rest condition (*p* < .001 uncorrected)

Region	Side	BA	MNI coordinates			*Z* value
			*X*	*Y*	*Z*	
Superior Parietal Lobule	R	7	28	−56	41	4.55
Precuneus	R	7	22	−59	46	4.02
Middle Frontal Gyrus	R	6	39	2	41	4
Precentral Gyrus	R	9	41	8	33	4.31
Precentral Gyrus	L	6	−42	−3	41	3.84
Culmen	R		10	−33	−4	4.27
Parahippocampal Gyrus	R	30	19	−33	−4	4.25
Superior Parietal Lobule	L	7	−24	−56	38	4.16
Superior Parietal Lobule	L	7	−22	−66	45	3.45
Precuneus	L	7	−15	−66	43	4.25
Inferior Parietal Lobule	R	40	34	−45	42	3.94
Insula	R	13	40	−26	4	3.76
Thalamus	R		25	−28	6	4.07
Declive	L		−31	−64	−11	4.05
Cingulate Gyrus	R	31	17	−34	28	3.93
Thalamus	R		6	−23	11	3.83
Declive	R		34	−67	−18	3.74
Culmen of Vermis	L		−1	−67	1	3.73
Precuneus	R	31	13	−59	31	3.71
Culmen	L		−14	−44	−6	3.69
Parahippocampal Gyrus	L	19	−20	−50	−3	3.52
Caudate	R		14	−1	19	3.5
Precuneus	R	7	13	−40	51	3.46
Fusiform Gyrus	R	37	38	−49	−11	3.43
Hippocampus	R		28	−36	3	3.43
Declive	L		−34	−76	−22	3.41
Thalamus	R		19	−8	13	3.41
Thalamus	L		1	−29	11	3.4
Subgyral	R	6	22	−5	53	3.33
Cingulate Gyrus	L	24	−16	2	42	3.3
Thalamus	L		−9	−17	10	3.29
Precuneus	R	7	11	−65	36	3.28
Declive	R		38	−58	−15	3.28
Caudate	R		12	−19	24	3.27
Precentral Gyrus	L	6	−33	1	34	3.27
Posterior Cingulate	R	29	17	−48	11	3.25
Cingulate Gyrus	L	31	−16	−40	27	3.25
Lentiform Nucleus—Putamen	R		27	−16	12	3.21
Superior Temporal Gyrus	R	22	49	−15	−2	3.2
Lateral Geniculum Body	R		21	−24	−3	3.19
Superior Temporal Gyrus	R	41	45	−34	9	3.17
Thalamus	L		−18	−27	18	3.17
Culmen	R		29	−50	−18	3.15
Thalamus	L		−25	−26	7	3.15
Thalamus	L		−25	−29	10	3.14
Anterior Cingulate	L	24	−8	31	18	3.14
Thalamus	L		−12	−18	20	3.13
Thalamus	R		21	−15	5	3.13
Medial Frontal Gyrus	L	8	−9	17	47	3.11

Abbreviation: MNI = Montreal Neurological Institute.

**Table 8:** Between‐group (MDD > BC) comparisons of the retrieval > rest condition (*p* < .001 uncorrected)

Region	Side	BA	MNI coordinates			*Z* value
			*X*	*Y*	*Z*	
Middle Frontal Gyrus	L	9	−36	21	27	4.31
Culmen	L		−29	−50	−17	3.37
Superior Frontal Gyrus	L	6	−7	11	52	3.12

Abbreviation: MNI = Montreal Neurological Institute.

## 4 DISCUSSION

The primary finding from our current study was that both obesity alone and obesity in conjunction with MDD were associated with distinct neural activation patterns. Importantly, the presence of common medical comorbidities, namely hypertension, T2D, hyperlipidemia, and OSA, was not significantly different across the two bariatric groups (BC and B‐MDD) and thus was unlikely to be driving the group differences between these two groups. The use of both 3‐group conjunction analyses as well as separate pair t‐contrast analyses allowed us to identify areas commonly activated across all three groups, while also investigating differences in regional activation.

During encoding, all three groups showed strong FWE‐corrected conjunction activations in areas known to be involved in language processing and working memory (IFG), as well as the posterior cingulate (an area important in emotion and memory), indicating the task succeeded in showing activation of encoding and emotional processing of word stimuli by subjects. An fMRI‐adapted version of the Warrington's RMT has only previously been employed in a study of individuals with schizophrenia and HCs (Hofer et al., 2003). Similar to Hofer et al. (2003), regional activations were seen in the prefrontal, inferior frontal gyrus, and anterior cingulate.

Differences emerged in patterns of regional activation when comparing the BC > HC and B‐MDD > BC t‐contrast activation. The BC > HC contrast indicated a pattern of activation focused on regions in the temporal gyrus (MTG and STG) known to play an important role in memory formation (Squire & Zola‐Morgan, 1991). The increased BOLD response seen in these temporal areas in bariatric controls compared to healthy controls during memory encoding may be indicative of some neural compensation mechanism engaged to maintain task performance relative to HCs. This is supported by several studies that have reported that increased BOLD response in temporal regions is employed to compensate for neural inefficiency seen as a consequence of aging (Cabeza, Anderson, Locantore, & McIntosh, 2002; Rypma & D'Esposito, 2000). Temporal areas are known to be vulnerable to structural changes and volumetric losses in both MDD and obesity (Carnell et al., 2012; Minke et al., 2020; Shinsuke et al., 2018; Stanek et al., 2011), and given that the RMT task relies on memory systems linked to temporal areas, increased engagement of temporal areas may have been required by BC and B‐MDD groups to achieve adequate behavioral performance on the task.

Interestingly, a differing pattern of regional activation was seen when contrasting B‐MDD subjects to BC subjects during encoding. This contrast instead indicated that B‐MDD subjects relied on greater engagement of the precuneus and cingulate gyrus (notably, anterior cingulate gyrus). Precuneus connections are widespread and involve higher association of cortical and subcortical structures, important in the integration of external and self‐generated information and higher‐order cognitive functions (Cavanna & Trimble, 2006). Moreover, the precuneus (in conjunction with the cingulate and prefrontal cortices) is involved with episodic memory retrieval tasks, including word retrieval (Cabeza et al., 2002; Sajonz et al., 2010). It may be that B‐MDD subjects are engaging further compensatory systems in order to maintain memory performance, involving the precuneus and cingulate gyrus, given that there were no behavioral differences in verbal memory task performance between groups. In other words, compensatory engagement of the precuneus and cingulate gyrus may have allowed B‐MDD patients to achieve the same behavioral results as BC subjects, at the expense of increased neural energy and the engagement of a broader distributed network. This is further supported by previous studies that have also found that memory performance was associated with increased neural activity (rather than decreased activity) in regions of interest, potentially indicating poor inhibition resulting in higher error rates (Minke et al., 2020).

When investigating retrieval memory processes during the word recognition task, all three groups exhibited engagement of the dorsolateral prefrontal cortex, an area known to be important in working memory and executive function (Kane & Engle, 2002). When comparing paired group differences (t‐contrasts), we again noted that a large number of regions showed increased activation in BCs as compared to HCs. The precuneus and cingulate cortex were once again among regions of increased activation. A similar pattern of activation associated with obesity has been reported in a study showing increased cerebral metabolism in the posterior cingulate gyrus in obese women following bariatric surgery (Marques et al., 2014).

When looking at differences in activation between the B‐MDD and BC groups during retrieval, we found increased activation in the MFG and SFG. These brain regions were also activated in both the 3‐group conjunction analysis and BC > HC contrast, indicating that B‐MDD patients may require increased (compensatory) activation in memory and language processing regions.

Support for the functional differences we have demonstrated is found in recent structural and functional MRI investigations of obese populations (Minke et al., 2020; Stanek et al., 2011; van Tol et al., 2012). Growing research indicates that obesity is associated with structural brain changes that may contribute to cognitive impairment (Gustafson, 2012; Gustafson, Lissner, Bengtsson, Björkelund, & Skoog, 2004; Pannacciulli et al., 2006). Smaller regional volumes were related to higher BMI in the frontal, temporal, and parietal cortices, cerebellum, and midbrain (Taki et al., 2008) in a study of 1,428 individuals aged 12 – 81. In this study, an association between higher BMI and smaller brain volume was found in males only (not females). However, Walther, Birdsill, Glisky, and Ryan (2010) and Walther et al. (2010) found that increased BMI was associated with decreased volumes of gray matter in frontal and temporal regions as well as the right cerebellar region in older females. Increased BMI was also associated with increased white matter volume in frontal, temporal, and parietal lobes. Interestingly, gray and white matter volumes predicted performance on measures of memory and processing speed, despite the absence of significant group differences in cognitive performance. More recently, recent studies have shown that structural alterations in both gray and white matter density across various brain regions may be recovered as early as 6 months following bariatric surgery (which results in a dramatic weight loss) (Minke et al., 2020).

Taken together, our results support our hypothesis that obesity alone and obesity with MDD are associated with different neural patterns of activation during both encoding and retrieval processes. Additionally, changes seen in the precuneus, cingulate gyrus, and inferior, middle, and superior frontal gyrus may represent a neural compensation mechanism, allowing subtle cognitive impairment to go undetected by traditional neuropsychological measures. Further work is required to investigate the potential mechanisms contributing to these changes. Moreover, the addition of a fourth group of individuals with MDD who are not obese could further elucidate the potential additive and independent effects of MDD and obesity on cognition. Lastly, it must be noted that years of education differed when comparing bariatric groups to controls. However, standardized measures of IQ (in our study, the WASI) were included in order to address any potential cognitive differences that may have been found due to baseline intelligence differences. The WASI IQ measure did not significantly differ across groups in our study. Thus, the memory differences found reflect a true memory performance difference and cannot be stated to be driven by differences in baseline intelligence. Further, years of education include all post‐secondary education, not differentiating the level of that education, and is not as strong a correlate for cognitive ability as baseline intelligence might be.

As our study is cross‐sectional, it cannot speak to the causality of the associations seen between obesity, depression, and neural activation patterns. That they exist, however, given the high comorbidity between MDD and obesity is intriguing. The increased weight gain associated with certain psychotropic medications and implementation of a weight monitoring system in the treatment of MDD should be considered by healthcare professionals. The cognitive impairment associated with MDD and obesity may be distinct, but additive, leading to overall increased impairment and reduced functional ability in psychiatric populations.

## 5 AUTHOR CONTRIBUTION.

MR, MM, and VT conceived the study. MR, GH, MM, and VT curated the data. MR, MM, BF, GH, and VT performed formal analysis. VT and MR acquired funding. MR participated in investigation. MR, MM, GH, BF, and VT contributed to methodology. VT administered the project. VT, MM, and GH collected the resources. GH, MR, and BF developed the software. MM, BF, GH, and VT supervised the study. MR, MM, BF, GH, and VT validated the study. MR, MM, BF, GH, and VT contributed to visualization. MR wrote the original draft of the manuscript. MR, MM, BF, GH, and VT wrote, reviewed, and edited the manuscript.

## CONFLICT OF INTEREST

The authors have no conflicts of interest to declare. BF had a research grant from Pfizer, unrelated to this work.

## Data Availability

The data that support the findings of this study are available from the corresponding author upon reasonable request. The data are not publicly available due to privacy or ethical restrictions.
